# The Prescription Pattern of Chinese Herbal Products Containing *Ginseng* among Tamoxifen-Treated Female Breast Cancer Survivors in Taiwan: A Population-Based Study

**DOI:** 10.1155/2015/385204

**Published:** 2015-03-01

**Authors:** Wei-Lung Hsu, Yueh-Ting Tsai, Chien-Tung Wu, Jung-Nien Lai

**Affiliations:** ^1^Institute of Traditional Medicine, School of Medicine, National Yang-Ming University, No. 155, Section 2, Li-Nong Road, Taipei 112, Taiwan; ^2^Department of Chinese Medicine, Taipei City Hospital, Yangming Branch, No. 105 Yusheng Street, Shilin District, Taipei City 111, Taiwan; ^3^Taiwan Association for Traditional Chinese Medicine of Family, 9F., No. 105 Yusheng Street, Shilin District, Taipei City 111, Taiwan

## Abstract

*Background.* The purpose of our study is to analyze the association between prescribed Chinese herbal products (CHPs) containing *Ginseng* and the risk of endometrial cancer among tamoxifen (TMX) users and to identify any possible interactive effects between *Ginseng* and TMX with respect to preventing the development of subsequent endometrial cancer in an estrogen-dependent breast cancer population in Taiwan. *Methods.* All patients newly diagnosed with invasive breast cancer receiving tamoxifen treatment from January 1, 1998, to December 31, 2008, were selected from the National Health Insurance Research Database. The usage, frequency of service, and CHP-*Ginseng* prescribed across the 30,556 TMX-treated breast cancer (BC) survivors were evaluated. Logistic regression was employed to estimate the odds ratios (ORs) for the utilization of CHP-*Ginseng*. Cox's proportional hazard regression was performed to calculate the hazard ratios (HRs) for endometrial cancer associated with *Ginseng* use among the TMX-treated BC cohort. *Results.* The HR for the development of endometrial cancer among breast cancer survivors who had ever taken *Ginseng* after TXM treatment was significantly decreased compared to those who never used CHP. *Conclusion.* A significant inhibitory relationship between *Ginseng* consumption and subsequent endometrial cancer less than 2 years after TMX treatment was detected among BC survivors.

## 1. Introduction

Tamoxifen (TMX) is type of selective estrogen receptor modulator (SERM) that acts as an estrogen competitive inhibitor and prevents the bindings of estrogen to estrogen receptors (ERs). Among women with ER+ (estrogen receptor positive) breast cancer, tamoxifen reduces the risk of recurrence and death when given as adjuvant therapy during the early stages of the disease and also can act as a palliative when individuals have metastatic disease [1–4]. However, previous reports have indicated that there is an increasing risk of a carcinogenic effect on uterine tissue and this has been found to be associated with longer length of TMX treatment [1, 5–11]. In addition, tamoxifen is known to induce depression, hot flushes, and/or uterine abnormalities [12]. These effects involve a complex interaction between cancer-related symptoms and cancer-related mood disorders [13]. The above effects are some of the reasons why women with breast cancer often use complementary and alternative medicine (CAM), including herbs, vitamins, homeopathic remedies, and Chinese herbal products (CHPs) [14, 15].

*Ginseng* has a long history of use as part of the traditional Chinese pharmacopoeia and is often used in combination with other herbs. These mixtures, prescribed by traditional medicine (TM) practitioners in Japan, South Korea, China, and Taiwan, form many carefully balanced prescriptions that are tailored to a range of different gynecological ailments. Our previous studies have shown that, among the top ten most frequently prescribed Chinese herbal products (CHPs) used for the treatment of breast cancer in Taiwan, six contained* Ginseng* [16]. Ginsenosides are steroid saponins found in extracts from* Ginseng*. Previous studies have revealed that 20S-protopanaxadiol (aPPD), a major gastrointestinal metabolic product of ginsenosides, is able to induce apoptosis in various types of tumor cells [17]. Furthermore, these products also compete with the estradiol (E2) for binding to ERs, which blocks E2-induced transcriptional activation and inhibits colony formation by endometrial cancer cells [18]. It therefore is reasonable to hypothesize that prescription of* Ginseng* to TMX users by TCM doctors might reduce the occurrence of subsequent endometrial cancer.

Individuals in Taiwan are free to choose from care offered by western medicine clinics or by TCM clinics. With an insured rate of 98% to 99%, the random sample that comprises the NHI Research Database (NHIRD) is representative of the general population of Taiwan and should allow a reasonably accurate assessment of the coutilization of TCM and modern medical resources in Taiwan. Therefore, the NHIRD provides an ideal platform for pharmacoepidemiological studies [19, 20].

The aim of our study was to measure the association between prescription of Chinese herbal products containing* Ginseng* and the risk of endometrial cancer among TMX users. Furthermore, we also want to identify any possible interactive effects that might occur between* Ginseng* and TMX in terms of preventing the development of subsequent endometrial cancer in an estrogen-dependent breast cancer population.

## 2. Materials and Methods

### 2.1. Data Resources and Study Sample

Our study program was approved by the Institutional Review Board of the Taipei City Hospital. It was designed as a population-based observational study that had the following aims. These were to investigate, between January 1, 1998, and December 31, 2008, the prevalence of use of CHPs containing* Ginseng* (CHP-*Ginseng*) among TMX-treated BC survivors and to identify any associations between having been prescribed a CHP-*Ginseng* and the occurrence of subsequent endometrial cancer in Taiwan. All raw data were obtained from the NHI reimbursement database. The relevant information from the NHIRD that was used in the study had been encrypted with respect to identification numbers for all beneficiaries; this dataset was transformed by and is maintained by the National Health Research Institutes (NHRI) of Taiwan [21, 22]. The NHIRD records contained demographic information, including age, sex, and clinical data, as well as all records of clinical visits and hospitalizations, including the prescription and dosage of either conventional medicine including generic and commercial brands of tamoxifen or CHP formulae and single herbs that contain* Ginseng* obtained by patients. The diagnoses were adopted in the NHIRD according to the International Classification of Diseases, Ninth Revision, Clinical Modification (ICD-9-CM) [23]. Under the restriction of Taiwan's NHI, the medical record of all tamoxifen-treated BC survivors leaving Taiwan who will not be able to visit clinics in person or who died from any diseases before either the diagnosis of endometrial cancer or the end of the study in which condition health service fee will be no longer covered by NHI will not be reachable in the present study.

**Figure 1:**
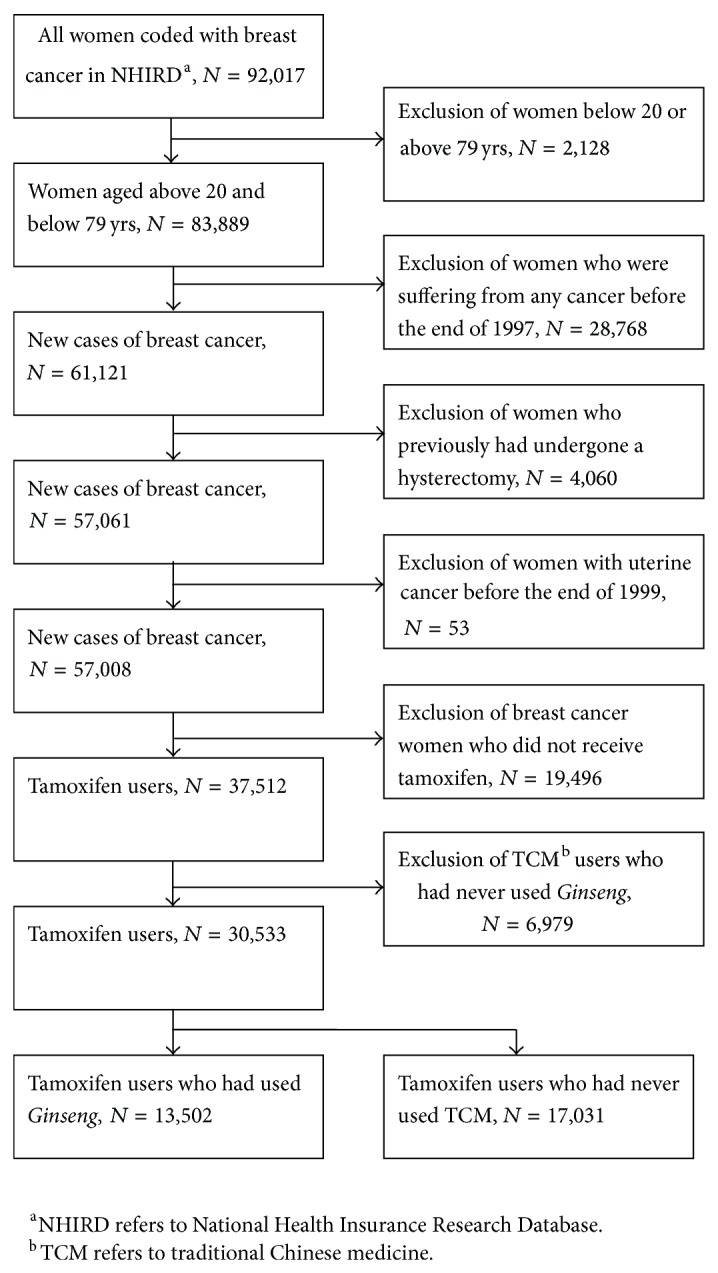
Algorithm describing the recruitment of study subjects from the National Health Insurance Catastrophic Illnesses Registry of Taiwan who were followed up from 1998 to 2008.

To select potential case subjects for this study, we screened the NHI Catastrophic Illness Registry files for all patients who were diagnosed as suffering from breast cancer from January 1, 1998, to December 31, 2008. Taiwan's NHI is a government-run, single-payer national health insurance scheme, financed on a pay-as-you-go basis through a mix of premiums and taxes. Therefore, all beneficiaries are designed to designate where the assets will go when they visit hospital or clinics in person or before they died. Because all patients who are registered as having a catastrophic illness are exempt from all copayments and must have been validated by tissue pathology and therefore this dataset is reliable and comprehensive. The selection of study subjects was performed as follows (Figure 1). Firstly, subjects under 20 or over 79 years of age (*n* = 2,128) were excluded to restrict the study sample to the adult cancer patients in Taiwan. Secondly, we excluded prevalent cases of cancer (including breast cancer) that had been diagnosed before the end of 1997; this was to ensure that the subjects were newly diagnosed with invasive breast cancer (BC survivors) during the period from 1998 to 2008 (*n* = 28,768). Furthermore, in order to control various potential confounding factors, we further excluded BC survivors who had not received tamoxifen treatment (*n* = 19,496) as well as any BC survivors who had undergone a previous hysterectomy (*n* = 4,060); this was to ensure that all the subjects included in this study were TMX-treated BC survivors. We identified all BC survivors 20 years of age or older who were continuously enrolled in the NHIRD during the years from 1998 to 2008. We recorded the starting and stopping dates and dosage for each period of tamoxifen treatment. The index date for given BC survivors was defined as the first date of filling a prescription for tamoxifen. Finally, in order to study the prescription pattern and effect of* Ginseng* only, we excluded all subjects who had been prescribed a TCM that did not contain* Ginseng* (*n* = 6,979). Finally, there were 30,533 TMX-treated BC survivors who could be divided into non-TCM users (*n* = 17,031) and* Ginseng *consumers (*n* = 13,502); these individuals formed the two cohorts that made up the study.

### 2.2. Traditional Chinese Medicine

There is a wide range of TCM treatments and these include the use of CHPs, acupuncture, and manual therapy; all of these are reimbursed by the NHI of Taiwan. CHPs consist of either a formula or a single herb and the use of CHPs is the most widely adopted TCM treatment in Taiwan [24]. To analyze the utilization of prescribed CHPs containing* Ginseng* in the present study, we gather information from the website of the Department of Chinese Medicine and Pharmacy, Ministry of Health and Welfare, Taiwan (DCMP). This information on CHPs includes the name of each herb present in all the various CHP formulae, the proportions of each herb in each CHP formula, the date and period of approval of the CHP, the name of the CHP manufacturer, and the CHP manufacturer's code [22].

### 2.3. Study Variables

To determine the key independent variables for the utilization of CHP containing* Ginseng *among TMX-treated BC survivors, we adopt a number of demographic factors that have been used by previous studies [16]. Ages were categorized into six groups, namely, 20–29, 30–39, 40–49, 50–59, 60–69, and 70–79 years. Geographic areas of residence of the subjects were divided into a number of different regions in Taiwan, namely, the northern region, the central region, the eastern region, the southern region, and the outlying islands. We also divided the subjects according to their monthly income into four ranks, namely, New Taiwan Dollars (NT$) 0, 1–19,999, 20,000–39,999, and ≥40,000. Furthermore, the duration of TMX treatment from the first date of filling a prescription for tamoxifen to diagnosis of endometrial cancer or the end of 2008 was categorized into four groups: <2 years, 2–4 years, 5–7 years, and ≥8 years. Cancer treatment modalities were divided into four groups, namely, TMX-alone, TMX plus surgery, TMX plus chemotherapy, and TMX plus surgery plus chemotherapy. Previous report indicated that endometrial cancer risk was elevated among BC survivors after tamoxifen treatment, for those with a history of high blood pressure and those with a history of diabetes [25, 26]. Furthermore, we also analyzed the risk of subsequent endometrial cancer between* Ginseng* users and non-TCM users after adjusting for age, the presence of diabetes mellitus, the presence of hypertension, the duration of TMX treatment (<2 years, 2–4 years, and >4 years), and the cumulative dose of TMX administered (<7,500 mg, 7,500–14,999 mg, 15,000–29,999 mg, and ≥30,000 mg).

### 2.4. Statistical Analysis

Descriptive statistics were used for the prescription rates of CHP-*Ginseng* users and these were stratified by the patient's age, the indications for the prescription, and the most frequently prescribed herbal formula. The main indications classification is based on the ICD-9 diagnosis of the patient. These ICD-9 diagnoses were grouped into various broad disease categories. Multiple logistic regression was conducted to evaluate the factors that correlated with* Ginseng* use based on odds ratios (ORs) and 95% confidence intervals (CIs). Within the study population, we identified 181 BC survivors who were newly diagnosed with subsequent endometrial cancer from January 1, 2000, to December 31, 2008, to allow at least 2 years between January 1, 1998, and the date of diagnosis of endometrial cancer to give sufficient time for case subjects to accumulate sufficient doses of tamoxifen to induce subsequent endometrial cancer. Eligible period was considered the period between either before the diagnosis of newly developed endometrial cancer or the end of the study and a BC survivor's first tamoxifen prescription. Cox's proportional hazard regression was performed to calculate the adjusted hazard ratio (adjusted HR) for subsequent endometrial cancer among the TMX-treated BC survivors who had used a CHP containing* Ginseng* compared with those who did not use any CHP. A significance level of *α* = 0.05 was selected. SAS statistical software package version 9.3 (SAS Institute, Cary, NC, USA) was used for all data management and analysis.

## 3. Results

The database of outpatient claims from 1998 to 2008 contained information on 30,533 TMX-treated BC survivors. Among these individuals, 13,502 (44.2%) breast cancer survivors used* Ginseng* during the study period (Table 1). Table 1 summarizes the characteristics of the study population, the number of incidents of endometrial cancer, the duration of TMX treatment, and cancer treatment modalities. The mean age of the non-TCM users was higher than that of the* Ginseng* users. There were more non-TCM users than* Ginseng* users with no income and who received TMX for less than 2-year duration. Within this population, we identified 181 patients who were diagnosed with subsequent endometrial cancer after TMX treatment (*Ginseng* users, *n* = 74; non-TCM users, *n* = 107) over the 11-year study period (1998–2008).

The adjusted odds ratios (aORs) and 95% confidence intervals (95% CIs) obtained by multiple logistic regression are summarized in Table 1. Compared with the age group 20–29 years (aOR = 1.00), there were significant differences between the* Ginseng* users and the non-TCM users aged 50 years and above in terms of their likelihood of using a TCM. Furthermore, TMX-treated BC survivors with higher incomes (NT$1–19,999: OR = 1.12; 95% CI: 1.05–1.19, NT$20,000–39,999: OR = 1.22; 95% CI: 1.13–1.32, and NT$ ≥40,000: OR = 1.32; 95% CI: 1.20–1.45) were more likely to be* Ginseng* users than were the patients with no income.

Analysis of the major disease categories affecting the 13,502* Ginseng* users (Table 2) showed that breast cancer was the most common reason for using a CHP containing* Ginseng* (30.06%, visits = 37,061), which was followed by “symptoms, signs, and ill-defined conditions” (24.06%, visits = 29,667) and “diseases of the digestive system” (11.61%, visits = 14,310); these results are summarized in Table 2. Details on the most frequently prescribed CHPs containing* Ginseng* by TCM doctors for treating BC survivors are provided in Table 3. As can be seen,* Tian Wang Bu Xin Dan* was the most frequently prescribed CHP containing* Ginseng*, followed by* Xiao Chai Hu Tang* and* Ban Xia Xie Xin Tang*.  Table 4 summarizes the different levels of risk with respect to endometrial cancer between* Ginseng* users and non-TCM users after different duration of TMX treatment (<2 years, 2–4 years, and >4 years) after adjusting for age, diabetes and hypertension, demographic variables, and cumulative TMX dose. We observed a negative relationship between taking* Ginseng* and endometrial cancer, which suggests that* Ginseng* may act as an inhibitor of carcinogenicity among TMX-treated BC survivors generally. Compared to non-TCM users, the adjusted HR for the development of endometrial cancer was decreased among* Ginseng* users by 0.59-fold (95% CI: 0.39–0.88) among the less than 2-year TMX treatment group, by 0.86-fold (95% CI: 0.47–1.56) among the 2–4-year TMX treatment group, and by 0.89-fold (95% CI: 0.39–2.04) among the longer than 4-year TMX treatment group. The adjusted HR magnitudes between the* Ginseng* users and non-TCM users aged 20–79 years who received less than 2 years of TMX treatment are consistent with the results obtained if we limit the analysis to postmenopausal women (55–79 years of age) only, although not significantly. However, in these circumstances, as shown in Table 5, the adjusted HR increased slightly among* Ginseng* users aged 55–79 years, although not significantly, if the BC survivors have received a longer duration of TMX treatment or have taken a cumulative TMX dose of more than 29,999 mg.

When the reference group consisted of BC survivors aged 55–79 years who had received less than 2 years of TMX treatment, the adjusted HRs were not statistically significantly increased for the 2–4-year TMX treatment group or for the longer than 4-year TMX treatment group. Furthermore, there was no significant effect of the cumulative TMX dose on the risk of developing subsequent endometrial cancer among the TMX-treated BC survivors aged 55–79 years when patients with larger cumulative doses were compared to patients with cumulative doses of less than 7,500 mg (Table 6).

## 4. Discussion

This population-based study is the first study to our knowledge to document the association between prescribed Chinese herbal products containing* Ginseng* and the risk of endometrial cancer among TMX users. Furthermore, it is also the first to investigate the possible interactive effects between* Ginseng* and TMX in terms of preventing the development of subsequent endometrial cancer among an estrogen-dependent breast cancer population. This study has a number of strengths that deserve attention. Firstly, the NHI reimbursement database collects all prescription information prospectively and therefore we can rule out the possibility of recall bias with respect to the intake of various Chinese herbal products. Secondly, in this study, we included all patients newly diagnosed with breast cancer in Taiwan from 1997 to 2008 and therefore we can also rule out the possibility of selection bias. Thirdly, in order to prevent potential confounding factors affecting our results with respect to TMX, we excluded subjects who had ever been prescribed TMX previously. In this study, we allowed a minimum induction time of 2 years and calculated the cumulative dose up to 2 years before diagnosis in order to detect most, if not all, cases of endometrial cancer. Moreover, to minimise other potential confounding factors, we also carried out an analysis specifically on postmenopausal women (55–79 years old) who are at the age of ovarian failure. Using this subgroup, the association between* Ginseng* consumption and the protective effects of subsequent endometrial cancer remains observable, as summarised in Table 5. Taken together, our findings show that there is a potential inhibition effect with respect to the development of endometrial cancer when a* Ginseng* containing CHP is prescribed in parallel with TMX treatment.

Although some studies in Western countries have observed a positive association among estrogen-positive breast cancer survivors between subsequent endometrial cancer and TMX exposure, there have been few reports in Asia that have tested this hypothesis. Due to a fear of challenge by the patient and/or her family, physicians in Taiwan typically prescribe TMX to any BC survivors as a precautionary measure if there is any doubt with regard to endometrial hyperplasia after TMX treatment. Under the NHI system in Taiwan, all individuals who receive TMX are required to undergo a follow-up by their physicians no longer than three months after the start of treatment. These guidelines increase the likelihood of detecting endometrial cancer at an earlier stage of disease rather than later during the invasive stage. Based on the above, it is reasonable that, among TMX-alone group, >60% of all subsequent endometrial cancers were found to be detected after a TMX exposure of less than 2-year duration or after cumulative doses of less than 7500 mg, as shown in Table 5. Another possible explanation for this early detection might be that the Asian women are generally slimmer than women from Western countries and we are unable to rule out the possibility that their exposure to TMX based on used guideline recommendations resulted in the development of subsequent endometrial cancer earlier and at small cumulative doses of TMX. Previous studies have indicated that TMX is a genotoxic carcinogen that may act as an initiator of cancer, which might subsequently lead to development of endometrial cancer. It is well known that endometrial cancer has a number of specific features, namely, single cell origin, irreversibility, and racial/ethnic differences; these make it difficult to determine the allowable upper limits of human exposure to TMX. The present findings, as shown in Table 6, corroborate the results of the previous reports, which have demonstrated that TMX treatment that is of longer duration and has higher cumulative doses does not seem to be associated with an increased risk for subsequent endometrial cancer. This observation warrants further studies that should determine whether there is a differential genetic susceptibility between Asian and Caucasian postmenopausal women.

One distinguishing feature of the national health care system in Taiwan is the coexistence of modern western medicine and TCM. Breast cancer survivors who perceive any cancer-related or cancer treatment-induced discomforts in Taiwan are free to choose from care offered by oncologists or by TCM clinics; this has led to up to 40% of BC survivors at some point or another consuming a CHP containing* Ginseng* during the study period. Specifically, those who took TMX for a longer period are more likely to be* Ginseng* consumers.* Ginseng* has a long history of use as part of the traditional Chinese pharmacopoeia and was first documented in the classical Chinese text* Shennong Bencao Jing* circa 100 A.D. by* Shennong*; this referenced* Ginseng*'*s* cooling and calming properties, its use to treat a poor nutritional status, and its ability to alleviate digestive distress [27].* Tian Wang Bu Xin Dan* was found in this study to be the most frequently prescribed formula containing* Ginseng* used by TMX-treated BC survivors in Taiwan, as is shown in Table 3.* Tian Wang Bu Xin Dan* has a long history of use as part of the traditional Chinese pharmacopoeia and was first documented as being able to replenish the blood, replenish* Yin*, and strengthen heart functions; this then leads to eventual relief of insomnia, heart palpitations, forgetfulness, and anxiety. Among the other nine most frequently prescribed formulae containing* Ginseng*, some are associated with relieving gastrointestinal discomfort (*Xiao Chai Hu Tang*,* Ban Xia Xie Xin Tang,* and* Xiang Sha Liu Jun Zi Tang*), others with relieving heart palpitations and fatigue (*Zhi Gan Cao Tang*), and yet others with relieving forgetfulness, tiredness, and breathing difficulties (*Bu Zhong Yi Qi Tang* and* Gui Pi Tang*), while others are associated with relieving musculoskeletal disorders (*Du Huo Ji Sheng Tang*), with restoring physical strength (*Sheng Mai San*), and with relieving shortness of breath and coughing (*Mai Men Dong Tang*). The high prevalence of* Ginseng* use probably is because TCM doctors intended to relieve patients' complaints in the areas of mental and physical efficiency, fatigue, gastrointestinal disorders, inability to concentrate, and irritability [28–30], all of which might be associated with TMX-induced side effects [7–12]. Further analysis found that, compared with those who received TMX-alone treatment, BC survivors who received optimal surgery plus TMX are more likely to be prescribed a CHP containing* Ginseng* by TCM doctors. The present results are in accordance with previous animal studies showing that the active ingredient of* Ginseng* might benefit patients who have received surgery with respect to healing [31], convalescence, and postoperative syndrome [32, 33]. TCM doctors gather clinical symptoms and signs via four specific diagnostic methods (inspection, listening and smelling, inquiry, and pulse-feeling and palpation) and from these decide on the* Qi* deficiency pattern of the patient, which refers to the body's state of health in this case at a certain stage after or under TMX treatment, which may be either TMX treatment alone or in conjunction with surgery. At this point, a treatment principle for supplementing or strengthening* Qi *is put forward in accordance with the particular pattern observed; this may result in a formula containing* Ginseng* being prescribed to adjust the disharmony in the functioning of* Qi* or the interactions involving* Qi*. The aforementioned procedure is one of most important fundamental characteristics of traditional Chinese medicine (TCM), which has a unique approach to the diagnosis and treatment of the essence of the disease pathological process. This approach differentiates TCM from western medicine. Interestingly, TCM doctors who prescribed a formula containing* Ginseng* to patients who had undergone TMX treatment do not with the intent of inhibiting endometrial cancer growth; thus, the resulting statistically significantly low risk of developing subsequent endometrial cancer was serendipitous. This interesting finding with respect to* Ginseng *implies that a combination of the practices and methods of traditional Chinese medicine with conventional biomedicine, with the former being focused on the wellness and health of the patient, while the latter is focused on treating the breast cancer, may be a more optimal approach when treating breast cancer survivors.

To our limited knowledge, the present study is the first of its kind to report an inhibitory relationship between* Ginseng* consumption and subsequent endometrial cancer among BC survivors who have undergone less than 2 years of TMX treatment. It is important to note that, among women with reproductive age, TMX has an antiestrogenic effect on the uterus, while, in contrast, among postmenopausal women, TMX has an estrogenic effect [34]. The reason why it is important to limit the analysis to postmenopausal women (55–79 years old) as part of the general analysis is that this minimizes any potential confounding effects that might be associated with the serum level of estrogen among the women of reproductive age. Again, just like the general population, there was an inhibitory effect of* Ginseng* on the development of subsequent endometrial cancer among* Ginseng* consumers in the less than 4-year duration group or in the lower than 29,999-mg cumulative dose group, although not statistically significant. However, before drawing any conclusions from these findings, further studies are needed among breast cancer survivors who have received long-term tamoxifen administration in order to analyze the level of repair in relation to major tamoxifen-DNA adducts both in the presence and in the absence of* Ginseng. *This is especially needed for postmenopausal women where the confounding effects of estrogen are absent.

The present study has a few limitations. Firstly, because the identities of the patients were encrypted and thus are not available within the NHI reimbursement database, we were unable to obtain any histopathological data to verify the patient's diagnosis. However, because approval is required for the registration of invasive breast cancer or endometrial cancer as a catastrophic illness and the fact that this needs to be based on pathological and/or cytological evidence, which is then followed by a full waiver of copayment, such a diagnosis is made only after very serious consideration and is highly likely to be very accurate. The diagnostic accuracy of invasive breast cancer among the NHI data is corroborated by the considerable agreement between the incidence rate calculated herein and those determined by the National Cancer Registry of Taiwan, in which 95% of the breast cancers are accompanied by histopathological validation. Secondly, this study did not include Chinese herbal remedies purchased directly from TCM herbal pharmacies, nor have we included health foods that may contain herbs. Thus, the frequency of* Ginseng* utilization might have been underestimated. However, because the NHI system has a comprehensive coverage for TCM prescriptions, the cost of which is generally less than the cost of herbs sold in Taiwan's markets, the likelihood that subjects purchased and consumed large amounts of other herbs outside the NHI database is not high. Thirdly, we were unable to validate the actual ingested dose of the prescribed TMX recorded in the database. This is because a large mean cumulative dose in this study indicates that the patient continued to receive the same prescription for a long period and thus this implies that the patient actually consumed the prescribed medication; however, this does prove consumption took place. Even if the patient did not consume all of the prescribed TMX, the present findings would only underestimate the effect of the consumed TMX. Fourthly, because the reimbursement data did not include the patterns of consumption of phytoestrogen-rich foods, the relative weight of the patients, or reproductive history of the patients, we were unable to control these factors as part of the model construction. Lastly, this study did not include tamoxifen-treated BC survivors leaving Taiwan or who died from any diseases before either the diagnosis of endometrial cancer or the end of the study.

## 5. Conclusions

A large proportion of TMX-treated BC survivors had at some point consumed CHPs containing* Ginseng* with the intention of relieving their breast cancer and tamoxifen-induced symptoms. A significant inhibitory relationship between* Ginseng* consumption and subsequent endometrial cancer among BC survivors was found; this trend was also observed for individuals aged 55–79 years with less than 4 years of TMX treatment. Further studies are warranted that analyze the repair of the major tamoxifen-DNA adducts and the additive effects that* Ginseng* seems to have on this repair, especially among women who have undergone ovarian failure.

## Figures and Tables

**Table 1:** Demographic characteristics and results of multiple logistic regression showing the adjusted odds ratios (aORs) and 95% CIs (confidence intervals) for tamoxifen-treated breast cancer survivors from the National Health Insurance Catastrophic Illnesses Registry of Taiwan who were followed up from 1998 to 2008.

Characteristics	Tamoxifen users who had ever used ginseng (%)	Tamoxifen users who had never used TCM^a^ (%)	aOR^b^ (95% CI^c^)
Number of cases	13,502	17,031	
Number of endometrial cancers	74	107	
Age at diagnosis of breast cancer			
Average	50.1 ± 10.8	52.4 ± 11.8	
20~29 yrs	193 (1.4)	194 (1.1)	1
30~39 yrs	1,896 (14.0)	1,987 (11.7)	0.87 (0.70–1.08)
40~49 yrs	5,099 (37.8)	5,711 (33.5)	0.81 (0.66–1.00)
50~59 yrs	3,551 (26.3)	4,468 (26.2)	0.75 (0.61–0.92)
60~69 yrs	2,005 (14.8)	2,903 (17.0)	0.65 (0.53–0.81)
70~79 yrs	758 (5.6)	1,768 (10.4)	0.43 (0.34–0.54)
Insured salary ($NT/month)			
0	2,337 (17.3)	3,636 (21.3)	1
1–19999	7,160 (53.0)	8,863 (52.0)	1.12 (1.05–1.19)
20000–39999	2,663 (19.7)	3,081 (18.1)	1.22 (1.13–1.32)
>=40000	1,342 (9.9)	1,451 (8.5)	1.32 (1.20–1.45)
Insured region			
Northern Taiwan	6,593 (48.8)	9,766 (57.3)	1
Central Taiwan	2,827 (20.9)	2,375 (13.9)	1.89 (1.77–2.01)
Southern Taiwan	3,643 (27.0)	4,323 (25.4)	1.28 (1.21–1.36)
Eastern Taiwan	287 (2.1)	354 (2.1)	1.23 (1.05–1.45)
Outlying islands	152 (1.1)	215 (1.3)	1.06 (0.86–1.32)
Duration of tamoxifen treatment			
<2 y	6,524 (48.3)	11,411 (67.0)	1
2–4 y	5,908 (43.8)	4,756 (27.9)	2.15 (2.05–2.26)
5–7 y	983 (7.3)	773 (4.5)	2.20 (1.99–2.44)
>=8 y	87 (0.6)	91 (0.5)	1.69 (1.26–2.28)
Cancer treatment modalities			
Tamoxifen only	190 (1.4)	386 (2.3)	1
Tamoxifen and chemotherapy	290 (2.1)	777 (4.6)	0.70 (0.56–0.87)
Tamoxifen plus surgery	3,514 (26.0)	4,569 (26.8)	1.21 (1.01–1.46)
Tamoxifen, surgery, plus chemotherapy	9,508 (70.4)	11,299 (66.3)	1.22 (1.02–1.47)

^a^TCM refers to traditional Chinese medicine; ^b^OR refers to odds ratio; ^c^CI refers to confidence interval.

**Table 2:** Frequency distribution of traditional Chinese medicine (TCM) with *Ginseng* visits by major disease categories (according to ICD codes) among tamoxifen-treated breast cancer survivors from the National Health Insurance Catastrophic Illnesses Registry of Taiwan who were followed up from 1998 to 2008.

Major disease category	ICD-9-CM code range	Visit	%
Neoplasms	140–239	39,089	31.7
(including breast cancer)	174	37,061	30.06
Symptoms, signs, and ill-defined conditions	780–799	29,667	24.06
Diseases of the digestive system	520–579	14,310	11.61
Diseases of the respiratory system	460–519	11,996	9.73
Diseases of the musculoskeletal system and connective tissue	710–739	8,935	7.25
Diseases of the genitourinary system	580–629	6,284	5.1
Diseases of the nervous system and sense organs	320–389	3,428	2.78
Diseases of the circulatory system	390–459	2,409	1.95
Injury and poisoning	800–999	1,984	1.61
Endocrine and metabolic diseases, immunity disorders	240–279	1,925	1.56
Others		3,278	2.7

Total		123,305	100

**Table 3:** Top ten herbal formulae that form the TCM prescriptions with *Ginseng* that were prescribed to tamoxifen-treated breast cancer survivors from the National Health Insurance Catastrophic Illnesses Registry of Taiwan who were followed up from 1998 to 2008.

Herbal formulae	English name	Frequency of prescriptions	Daily dose of ginseng/prescriptions (g)^*^	Duration/prescriptions (day)
*Tian Wang Bu Xin Dan *	Celestial Emperor Heart-Supplementing Elixir	9,690	1.2 ± 0.8	9.2 ± 6.2
*Xiao Chai Hu Tang *	Minor Bupleurum Decoction	9,661	1.4 ± 1.1	7.6 ± 4.3
*Ban Xia Xie Xin Tang *	Pinellia Heart-Draining Decoction	9,655	1.8 ± 1.3	7.6 ± 4.7
*Xiang Sha Liu Jun Zi Tang *	Costus Root and Amomum Six Gentlemen's Decoction	9,406	1.0 ± 0.6	9.0 ± 5.5
*Zhi Gan Cao Tang *	Honey-Fried Licorice Decoction	8,584	0.4 ± 0.2	8.4 ± 4.9
*Bu Zhong Yi Qi Tang *	Center-Supplementing *Qi*-Boosting Decoction	8,327	1.4 ± 1.0	8.8 ± 5.4
*Gui Pi Tang *	Spleen-Returning Decoction	8,219	1.2 ± 0.9	9.6 ± 6.1
*Du Huo Ji Sheng Tang *	Pubescent Angelica and Mistletoe Decoction	7,238	0.9 ± 0.7	8.6 ± 5.7
*Sheng Mai San *	Pulse-Engendering Powder	7,172	5.8 ± 4.3	8.8 ± 4.8
*Mai Men Dong Tang *	Ophiopogon Decoction	6,532	1.2 ± 0.9	6.6 ± 3.1

^*^Presented as raw weight of ginseng.

**Table 4:** Number of new cases, population at risk, estimated hazard ratios (HR), and 95% confidence intervals (CI) for endometrial cancer estimated using the multivariate Cox's regression model from the National Health Insurance Catastrophic Illnesses Registry of Taiwan for all tamoxifen-treated breast cancer women 20–79 years who were followed up from 1998 to 2008.

Presence of endometrial cancer during the follow-up period	TMX users using Chinese medicine containing ginseng. Number of cases/person-years	TMX users not using Chinese medicine. Number of cases/person-years	TMX users using Chinese medicine containing ginseng divided by TMX users not using Chinese medicine. Adjusted HR^*^ (95% CI)
Total TMX users	**74/78,257**	**107/67,503**	**0.68 (0.51–0.93)**
Duration of TMX treatment			
<2 years	35/31,974	77/35,889	0.59 (0.39–0.88)
2–4 years	25/24,195	20/16,771	0.86 (0.47–1.56)
>4 years	14/22,088	10/14,842	0.89 (0.39–2.04)
Cumulative TMX doses			
<7,500 mg	19/18,630	62/24,030	0.47 (0.28–0.79)
7,500–14,999 mg	16/13,235	14/11,636	0.91 (0.44–1.90)
15,000–29,999 mg	24/23,890	21/16,581	0.79 (0.43–1.43)
≥30,000 mg	15/22,502	10/15,257	1.04 (0.46–2.35)

^*^Adjusted for age at breast cancer diagnosis, insured salary, insured region, diabetes, hypertension, duration of TMX treatment, and cumulative TMX doses.

TMX refers to tamoxifen.

**Table 5:** Number of new cases, population at risk, estimated hazard ratios (HR), and 95% confidence intervals (CI) for endometrial cancer estimated using the multivariate Cox's regression model from the National Health Insurance Catastrophic Illnesses Registry of Taiwan for all tamoxifen-treated breast cancer women 55–79 years old and followed up from 1998 to 2008.

Presence of endometrial cancer during the follow-up period	TMX users using Chinese medicine containing ginseng. Number of cases/person-years	TMX users not using Chinese medicine. Number cases/person-years	TMX users using Chinese medicine containing ginseng divided by TMX users not using Chinese medicine. Adjusted HR^*^ (95% CI)
Total TMX users	**30/25,206**	**47/27,681**	**0.75 (0.47–1.19)**
Duration of TMX treatment			
<2 years	10/10,379	33/15,576	0.50 (0.24–1.01)
2–4 years	12/7,396	11/6,370	0.93 (0.40–2.14)
>4 years	8/7,431	3/5,736	1.97 (0.51–7.66)
Cumulative TMX doses			
<7,500 mg	7/6,293	26/10,695	0.51 (0.22–1.17)
7,500–14,999 mg	4/4,051	7/4,868	0.55 (0.16–1.91)
15,000–29,999 mg	10/7,335	11/6,270	0.77 (0.32–1.84)
≥30,000 mg	9/7,527	3/5,848	2.44 (0.64–9.31)

^*^Adjusted for age at breast cancer diagnosis, insured salary, insured region, diabetes and hypertension, duration of TMX treatment, and cumulative TMX doses.

TMX refers to tamoxifen.

**Table 6:** Number of new cases, population at risk, estimated hazard ratios (HR), and 95% confidence intervals (CI) estimated using the multivariate Cox's regression model for a random sample from the National Health Insurance Research Database that included tamoxifen users stratified by age who were followed up from 1999 to 2008.

TMX use at baseline	TMX users using Chinese medicine containing *Ginseng* aged 55 to 79 years		TMX users not using Chinese medicine aged 55 to 79 years	
	Number of cases/person-years	Adjusted HR^*^ (95% CI)	Number of cases/person-years	Adjusted HR^*^ (95% CI)
Duration of TMX treatment				
<2 years	10/10,379	1	33/15,576	1
2–4 years	12/7,396	6.03 (0.72–50.62)	11/6,370	1.13 (0.12–10.66)
>4 years	8/7,431	2.68 (0.18–40.90)	3/5,736	0.67 (0.02–19.90)
Cumulative TMX doses				
<7,500 mg	7/6,293	1	26/10,695	1
7,500–14,999 mg	4/4,051	0.72 (0.19–2.81)	7/4,868	0.55 (0.24–1.28)
15,000–29,999 mg	10/7,335	0.22 (0.02–2.25)	11/6,270	0.65 (0.07–6.34)
≥30,000 mg	9/7,527	0.35 (0.02–5.60)	3/5,848	0.34 (0.01–10.36)

^*^Adjusted for age at breast cancer diagnosis, insured salary, insured region, diabetes and hypertension, duration of TMX treatment, and cumulative TMX doses.

TMX refers to tamoxifen.

## Abbreviations

TCM: Traditional Chinese medicine
CHPs: Chinese herbal products
TMX: Tamoxifen
SERM: Selective estrogen receptor modulator
BC: Breast cancer
ER: Estrogen receptors
NHIRD: National Health Insurance Research Database
ORs: Odds ratios
HR: Hazard ratio
CIs: Confidence intervals
CAMs: Complementary and alternative medicines
NHRI: National Health Research Institutes
ICD-9-CM: International Classification of Diseases, Ninth Revision, Clinical Modification
NHI: National health insurance.
